# Probiotics in Celiac Disease

**DOI:** 10.3390/nu10121824

**Published:** 2018-11-23

**Authors:** Fernanda Cristofori, Flavia Indrio, Vito Leonardo Miniello, Maria De Angelis, Ruggiero Francavilla

**Affiliations:** 1 Paediatric Department, “SS Annunziata” Hospital, 74100 Taranto, Italy; fernandacristofori@gmail.com; 2 Department of Paediatrics, Paediatric Hospital Giovanni XXIII, Via Amendola 207, 70126 Bari, Italy; flaviaindrio1@gmail.com (F.I.); vito.miniello@libero.it (V.L.M.); 3 Department of Soil, Plant and Food Sciences, University of Bari Aldo Moro, 70126 Bari, Italy; maria.deangelis@uniba.it; 4 Pediatric Section, Department of Interdisciplinary Medicine, University of Bari Aldo Moro, 70124 Bari, Italy

**Keywords:** probiotics, microbiota, celiac disease, gluten free diet

## Abstract

Recently, the interest in the human microbiome and its interplay with the host has exploded and provided new insights on its role in conferring host protection and regulating host physiology, including the correct development of immunity. However, in the presence of microbial imbalance and particular genetic settings, the microbiome may contribute to the dysfunction of host metabolism and physiology, leading to pathogenesis and/or the progression of several diseases. Celiac disease (CD) is a chronic autoimmune enteropathy triggered by dietary gluten exposure in genetically predisposed individuals. Despite ascertaining that gluten is the trigger in CD, evidence has indicated that intestinal microbiota is somehow involved in the pathogenesis, progression, and clinical presentation of CD. Indeed, several studies have reported imbalances in the intestinal microbiota of patients with CD that are mainly characterized by an increased abundance of *Bacteroides* spp. and a decrease in *Bifidobacterium* spp. The evidence that some of these microbial imbalances still persist in spite of a strict gluten-free diet and that celiac patients suffering from persistent gastrointestinal symptoms have a desert gut microbiota composition further support its close link with CD. All of this evidence gives rise to the hypothesis that probiotics might play a role in this condition. In this review, we describe the recent scientific evidences linking the gut microbiota in CD, starting from the possible role of microbes in CD pathogenesis, the attempt to define a microbial signature of disease, the effect of a gluten-free diet and host genetic assets regarding microbial composition to end in the exploration of the proof of concept of probiotic use in animal models to the most recent clinical application of selected probiotic strains.

## 1. Introduction

Celiac disease (CD) is a lifelong immune mediated enteropathy initiated by exposure to dietary gluten in individuals carrying human leucocyte antigen (HLA)-DQ2 or DQ8 [1]. Loss of gluten tolerance may occur at the time of its introduction into the diet or at any time in life, and the underlying mechanism is still under research. The role for an environmental component in CD pathogenesis is supported by: (a) HLA and non-HLA genes explain only 55% of disease susceptibility, (b) the concordance of celiac disease in monozygotic twins is around 80%, and (c) the incidence on this condition is rapidly increasing [2,3,4].

Intestinal microbiota could be somehow involved in the pathogenesis of CD and/or in its progression and/or in the development of clinical manifestation [5,6,7,8,9]. Briefly, gut microbiota can impact on the pathogenesis of CD in different ways: (a) modulating the digestion of gluten peptides both generating toxic and/or tolerogenic peptides that might impact on the acquisition of dietary tolerance to antigen, (b) influencing the intestinal permeability through zonulin release and tight junction expression, (c) promoting the maturation of the mucosal epithelium, and (d) regulating the activity of the immune system via expression of cytokines and pro-inflammatory or anti-inflammatory peptides [10].

In the last decade, several studies have reported imbalances in the intestinal microbiota of patients with CD, even though the literature shows that there is not a univocal microbial signature of CD [11]. It is also matter of debate whether dysbiosis plays a role in the pathogenesis of the disease, or whether it is just a consequence of CD inflammation; however, the intestinal dysbiosis often persists irrespective of the adherence to a gluten-free diet (GFD), and in part is also related to this particular diet. Finally, the identification of intestinal dysbiosis in CD, with the evidence supporting a role for gut microbiota in regulating key aspects of innate and adaptive immunity and the persistence of dysbiosis despite a prolonged GFD, have led to a hypothesis suggesting the clinical use of probiotics.

The aim of this review is to describe the recent scientific evidence on the role of gut microbiota in CD, and the proof of concept for the use of probiotics in CD patients.

## 2. Gut Microbiota and Risk of Developing Celiac Disease

Microbiota has a crucial role in the maturation of the immune system, being pivotal for the development of protective/tolerogenic immune responses [12]. Current evidence shows that environmental agents and/or endogenous signals may cause dysbiosis, which is responsible for a breakdown of immune homeostasis and an increase in the risk of immune conditions such as CD, among others [13].

There are several early life events that may prime a dysregulated gut microbiota, starting from the mode of delivery. After vaginal delivery, the colonization of the newborn is characterized mainly by *Lactobacilli*, *Prevotella,* and *Bifidobacteria* [14,15], while after cesarean section (C-section), the infant flora is mainly influenced by environmental and maternal skin bacteria [16]. This might explain an increased risk of CD in C-section newborns, as reported by previous studies [17,18].

Breastfeeding is a second factor that might impact gut microbiota composition; indeed, the presence of human maternal oligosaccharides supports the survival and growth of a healthy microbiota. Retrospective studies have shown that the duration of breastfeeding and particularly gluten introduction during breastfeeding reduce or delays CD onset [19]. However, both these evidence have been recently questioned and not confirmed, so the issue is still debated [20,21,22], and the issue may be more complicated than initially thought. De Palma et al. studied 164 newborns (born in a family with a first-degree relative with CD) divided according to HLA genotype and modality of feeding (breast versus formula), and found a different gut colonization according to with the type of feeding. Overall, they showed that carrying the HLA predisposition was associated with increased numbers of *Bacteroides fragilis* and *Staphylococcus*, and decreased *Bifidobacterium*, and that these differences were increased by formula as compared to breastfeeding. These results support the idea that gut microbiota composition is a multiplayer game where both feeding type and HLA genotype are key regulators [23]. Another variable can complicate this issue: evidence that breast milk samples from mothers with CD as compared to those without CD have lower titers of interleukin12p70, transforming growth factor-β1, and secretory immunoglobulin A (IgA), and a decrease in the *Bifidobacterium* and *Bacteroides fragilis* groups. This study supports the hypothesis that the reduction of immune-protective compounds and *Bifidobacterium* species can reduce the protection conferred by breastfeeding, thus increasing the child**’**s risk of CD [24]. 

That a particular genetic asset could play a role in shaping gut microbiota in early life is further supported by a recent study. De Palma et al. studied the faecal microbiota of 22 breastfed infants (born in a family with a first-degree relative with CD), and found that carrying a high (HLA-DQ2) as compared to a low genetic risk (non-HLA-DQ2/8) was followed by the presence of higher proportions of Firmicutes and Proteobacteria (*Corynebacterium, Gemella,* unclassified *Clostridiaceae,* unclassified *Enterobacteriaceae,* and *Raoultella*) and lower proportions of *Actinobacteria* (*Bifidobacterium* and unclassified Bifidobacteriaceae). These results highlight that a specific host genotype might modulate the gut microbiota composition of infants and contribute to an increasing disease risk [25]. The possibility that a particular genotype can shape the gut microbial composition is supported by genome-wide association studies that have identified 39 non-HLA CD risk loci. Interestingly, some of these genes related to immune functions and bacterial colonization and disease-associated single nucleotide polymorphism (SNPs) involved in the regulation of microbiota handling may explain the role of genes in gut microbiota composition [26].

In order to investigate the role of gut microbiota (and their products—metabolome) as contributory factors leading to the onset of CD, a large international study: “Celiac Disease Genomic, Environmental, Microbiome, and Metabolomic Study (CDGEMM) is ongoing in the United States (USA), Italy and Spain. CDGEMM is a prospective, longitudinal observational cohort study of infants with a first-degree family member with CD that aims to investigate if the time of gluten introduction, microbiota composition, and genetic asset are involved in the loss of gluten tolerance, and identify and validate specific microbiota and metabolic profiles that are mechanistically linked to gut functions (including permeability, immune function, and stem cell niche biology) and can anticipate a loss of gluten tolerance in genetically predisposed individuals. This study will be the proof of concept to plan preventive interventions to induce gluten immune tolerance and possibly prevent CD [27].

## 3. Microbiota in Celiac Patient

As shown in Table 1, in the last 10 years, several studies [28,29,30,31,32,33,34,35,36,37,38,39,40,41,42,43,44,45,46,47,48,49,50,51] have been performed evaluating fecal, salivary, and duodenal microbiota in CD patients. Interestingly, Collado et al. have shown a correlation between bacterial species found in both biopsies and feces of CD patients indicating that the fecal microbiota is comparable to the small intestine microbiota, and may have a diagnostic value [31].

Among the various studies, results may vary, which is due to huge differences in terms of microbiological methods, sample sizes, and patients’ characteristics. Nevertheless, there is substantial agreement on the presence of an imbalance between pro-inflammatory and anti-inflammatory species, with a prevalence of the former. 

We investigated the fecal microbiota of children with active CD (A-CD) and after (T-CD) GFD and of healthy children (HC) showing a reduction of *Lactobacill*us in A-CD, but not in T-CD, that was similar to that of HC. Using gas chromatography mass spectrometry solid-phase microextraction analysis, we found a profound variation of the mean concentrations of volatile organic compounds with short chain fatty acids being more represented in HC [32]. In a subsequent study, we analyzed the duodenal microbiota of 19 T-CD and 15 HC, and found a higher diversity of *Eubacteria* and lower counts of *Bifidobacteria* in T-CD as compared to HC children. According to the most recent scientific evidences, the CD patients’ microbiota seems to be characterized by an increased abundance of *Bacteroides* spp., *E. Coli*, *Proteobacteria,* and *Staphylococcus* and a decrease in *Bifidobacterium* spp. and *Lactobacillus* [52]. This result supports the knowledge that a long-lasting GFD did not completely restore the microbiota of CD children [37]. 

A study by Wacklin et al. suggested that the microbiota might have a role in the clinical manifestation of the disease. Indeed, the authors demonstrated that CD patients with gastrointestinal symptoms compared to those without and controls have different microbiota compositions (more abundant in *Proteobacteria* phylum versus more abundant in *Firmicutes* phylum, respectively) [44]. Moreover, alterations of microbiota may have pathogenic implication, leading to persistent gastrointestinal symptoms, despite a strict GFD. Indeed, the same group found that CD patients on a GFD who are still symptomatic have a reduced microbial richness and a different duodenal microbiota colonization in comparison with asymptomatic patients (higher relative abundance of *Proteobacteria* and a lower abundance of *Bacteroidetes* and *Firmicutes*), showing that intestinal dysbiosis might be responsible for the persistence of symptoms, even while adhering to a strict GFD [47].

## 4. Gluten-Free Diet and Gut Microbiota

At present, a strict GFD is the only available treatment [53] and, although evidence exists on the comparison between the gut microbiota of CD patients on a GFD or a gluten-containing diet (GCD) and/or controls, very few data are available in prospectively followed CD patients before and after GFD.

GFD is only partially effective in restoring the gut microbiota: indeed, while higher numbers of *Enterobacteria* or *Staphylococci* are restored, other alterations such as decreased *Bifidobacteria* and *Lactobacilli* and increased *Bacteroides*, *Enterobacteriaceae* and virulent *E. coli* still are persistent [54].

On the other hand, a GFD can itself influence gut microbiota composition. De Palma et al. studied the effects of a month of GFD on the composition of the gut microbiota in 10 healthy subjects, and found a significant decrease of *Bifidobacterium*, *Clostridium lituseburense,* and *Faecalibacterium prausnitzii,* and an increase of Enterobacteriaceae and *Escherichia coli* counts [54]. The analysis of the daily energy and nutrient intake before and after the GFD found no significant differences in dietary intake, except for a significant reduction in polysaccharide intake, leading the authors to conclude that a natural reduction in polysaccharide intake (fructans), which have prebiotic action and constitute one of the main energy sources for commensal components of the gut microbiota [55], might explain the reductions in beneficial gut bacteria populations. Therefore, a GFD itself rather than CD may be responsible for gut microbiota unbalance.

## 5. Probiotics Supplementation

Most of the evidence on the effect of probiotics in CD comes from animal models. Experiments using transgenic non-obese diabetic-DQ8 mice are the proof of concept that the microbiota shape the gluten-related immune-mediated mucosal damage. In germ-free conditions, mice develop a more aggressive gluten-induced pathology as compared with mice colonized with altered Schaedler flora (benign microbiota) that is deprived of opportunistic pathogens. However, in the presence of a microbiota with opportunistic pathogens or in the case of perturbations secondary to antibiotic use, mice develop gluten-induced severe pathology. These results reinforce the pivotal effect of gut microbiota in the inflammatory response that is associated with gluten ingestion [56]. 

Mouse models have demonstrated that probiotics can modulate innate and adaptive immunity, and reduce gliadin-induced inflammation [57,58,59].

Lindfors K et al. studied whether *Lactobacillus fermentum* or *Bifidobacterium lactis* are able to reduce the toxic effects of gluten-derived peptides in intestinal cell culture (Caco-2) conditions. They showed that *Bifidobacterium lactis* was able to inhibit the gliadin-induced derangement of epithelial permeability, and speculated that this probiotic could counteract the harmful effects of toxic gliadin epitopes [60].

Papista C et al. investigated the influence of probiotics in a model of gluten sensitivity (BALB/c mice); the authors were able to show that the *Saccharomyces boulardii KK1* strain hydrolyzed the gliadin toxic peptides, and its consumption was followed by improved enteropathy and a decrease of histological damage and pro-inflammatory cytokine production [59].

Laparra J.M. et al. studied the use of *Bifidobacterium longum CECT 7347* in an animal model of gliadin-induced enteropathy. The authors showed that the administration of this particular strain reduces the production of pro-inflammatory cytokines and the mediated immune response [61].

The idea that the effect played by probiotics is strain-specific is supported by the work of D’Arienzo et al., who studied the effect of *Lactobacillus* and *Bifidobacterium lactis* strains in transgenic mice expressing human DQ8, and found an increased antigen-specific tumor necrosis factor (TNF) secretion showing that probiotics may have pro-inflammatory rather than suppressive effects [62].

Despite the encouraging data deriving from in vitro studies, few in vivo data are available on probiotics supplementation in patients with CD (Table 2).

Smecuol et al. investigated the effects of *Bifidobacterium infantis* Natren life start strain (NLS-SS), randomizing 22 patients with A-CD to receive the probiotic or placebo while on a GCD, showing that this probiotic led to a significant improvement in GI symptoms. However, they found no effect on cytokines and growth factors, neither on celiac serology nor gut permeability [63].

The same group speculated that the favorable effect that was observed could be due to its influence on innate immunity. Thus, they tested the effect of *Bifidobacterium infantis* NLS-SS by assessing Paneth cells and macrophage counts and human α-defensin 5 (HD5) expression in duodenal biopsies of CD patients on a GFD. The results of this second study demonstrated that patients that assumed *Bifidobacterium infantis* NLS-SS experience a decrease in the expression of the antimicrobial peptide HD5, which is paralleled by a decrease in Paneth cells counts [64]. 

In a recent randomized control trial, Olivares at al. demonstrated in children with a new diagnosis of CD that the administration of *Bifidobacterium longum* CECT 7347 for three months, when associated with a GFD, was able to determine a height percentile increase compared with a placebo, as well as lower peripheral CD3^+^ T lymphocytes concentration and slightly reduced TNF-α levels; moreover, the treatment with *Bifidobacterium longum* CECT 7347 was associated with a significant decrease in the *Bacteroides fragilis* group and *Enterobacteriaceae* and a higher ratio of harmless to potentially harmful bacteria. However, the authors did not find any improvement of gastrointestinal symptoms [65].

Quagliarello et al. performed a RCT in 49 CD children to evaluate the efficacy of three months of administration of two *Bifidobacterium breve* strains (B632 and BR03) on the re-establishment of eubiosis in CD children on a GFD, demonstrating that supplementation induces an increase of Actinobacteria as well as a restoration of the Firmicutes/Bacteroidetes ratio [50]. 

On the contrary, Harnett et al. randomized 45 CD patients on a GFD, with persistent symptoms, to receive VSL#3 (5 g) or placebo, and found no differences in the fecal microbiota counts, and symptoms severity after two weeks of supplementation [66]. 

Klemenak et al. investigated the effect of two *Bifidobacterium breve* strains (BR03 and B632) on serum interleukin-10 and TNF-α levels in 49 children with CD on GFD, demonstrating lower levels of TNF-α after three months of daily use; no difference was found for interleukin (IL)-10 levels [67]. 

In 2018, Primec M. et al. performed a double-blind placebo-controlled study enrolling 40 CD and 16 healthy children. CD children were randomized to receive placebo or a mixture of two *Bifidobacterium breve* strains (DSM 16604 and DSM 24706) for three months. The authors showed that this probiotic mixture was able to modulate the production of acetic acid and total short-chain fatty acids (SCFAs), promoting a potential role in microbiome restoration [68].

Finally, our group recently performed a large prospective, randomized study in 109 CD patients strictly adherent to a GFD with irritable bowel syndrome (IBS) symptoms. Enrolled patients were randomized to probiotics (mixture of five strains of lactic acid bacteria and *bifidobacteria*: *Lactobacillus casei* LMG 101/37 P-17504 (5 Å~109 CFU/sachet), *Lactobacillus plantarum* CECT 4528 (5 Å~109 CFU/sachet), *Bifidobacterium animalis* subsp. *lactis* Bi1 LMG P-17502 (3.4 Å~109 CFU/sachet), *Bifidobacterium breve* Bbr8 LMG P-17501 (3.4 Å~109 CFU/sachet), *Bifidobacterium breve* Bl10 LMG P-17500 (3.4 Å~109 CFU/sachet)), or placebo for six weeks, and then followed up for six more weeks. Our results showed that the probiotic mix under study is effective in ameliorating the severity of IBS symptoms measured by IBS severity score (IBS-SS). After six weeks of treatment, we found a significantly higher proportion of treatment success (a decrease of at least 50% of IBS-SS), at both intention-to-treat (14.8% versus 3.6%; *p* < 0.04) and per protocol analysis (15.3% versus 3.8%; *p* < 0.04) [69]. A recent meta-analysis has shown that CD patients with GI symptoms have a higher prevalence of small intestinal bacterial overgrowth (SIBO) as compared to controls (28% versus 10%), although the difference does not reach statistical significance, and the analysis is affected by the large heterogeneity of the studies [70]. At present, no studies have been conducted to investigate whether probiotic administration might have an impact on SIBO in CD patients, nor have we explored this in our trial. However, we were able to show a positive modulation of gut microbiota with an increase of *bifidobacteria* still detectable six weeks after the discontinuation of probiotics [69]. 

## 6. Conclusions

**Figure 1:**
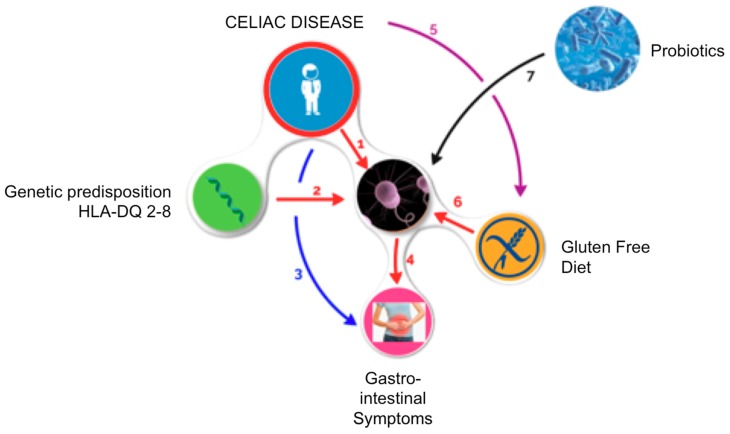
Mechanism of action of probiotics in controlling GI symptoms in celiac patients. Recent data have shown that patients with celiac disease (CD) have an altered gut microbiota (GM), (1) and that carrying the genetic predisposition (HLA-DQ-2 or DQ-8) may predispose individuals to a state of dysbiosis. (2) Patients with CD usually have GI symptoms (3) that can persist to a strict gluten-free diet (GFD); moreover, the alteration of GM can be one of the main causes of the persistence of GI symptoms. (4) CD requires that a patient follow a rigorous GFD (5) and a natural reduction in polysaccharide intake (fructans), which have prebiotic action, and constitute one of the main energy sources for commensals of the GM that might further worsen gut dysbiosis. (6) In turn, this reinforces the persistence of GI symptoms. (7). If we consider that most of the variables of this complex equation are fixed (genetic predisposition, CD, need for a GFD, the presence of GI symptoms), the only variable on which we can operate is the GM: therefore, the adoption of a probiotic supplementation that restores the imbalance in the GM of a celiac patient might be a reasonable therapeutic option.

Gut microbiota is an essential mediator of health, and its imbalance might be followed by an alteration of microbiota functions with a negative impact on health. Research in the last 10 years has shed new light on the role of the gut microbiota in CD and the complex relation between its composition, genetic background, GFD, and the persistence of clinical symptoms. Although many critical issues remain to be defined, some aspects are now clear. (a) Gut microbiota participate and mediate the gluten related inflammation. (b) As of yet, there is not a definite microbial signature of disease, although some microbial alterations are consistently reported, both in biopsies and fecal samples (abundance of *Bacteroides* spp., a decrease in *Bifidobacterium* spp.). (c) Some alterations of gut microbial composition revert to normal, while others are sustained by a GFD, and might be in part responsible for the persistence of symptoms in this population. (d) Selected probiotics with clinical proven efficacy might be of help in controlling gluten-mediated inflammation and ameliorating clinical symptoms (Figure 1).

With the increasing prevalence of people that adopt the gluten-free regimen, it is mandatory to define the intimate link between gut microbiota and gluten-related disorders in order to explore new possible avenues to offer a valid dietetic counseling to this expanding population and possibly in the future to identify new strategies for prevention and treatment.

## Figures and Tables

**Table 1:** Scientific findings of the last 10 years on salivary, duodenal, and fecal microbiota in celiac patients.

Author	Population	Age	Saliva Samples	Duodenal Biopsies	Fecal Samples	Methods	Results in CD Patients
Collado et al. [28]	26 CD vs. 23 HC	Children	No	No	Yes	Colture and FISH	↑ *Bacteroides–Prevotella, Clostriudium hystoliticum, Eubacterium rectale–C. coccoides, Atopobium and Staphylococcus*
Sanz et al. [29]	10 CD vs. 10 HC	Children	No	No	Yes	Culture DGGE	*L. curvatus, Leuconostoc mesenteroides* only in CD
Nadal et al. [30]	20 CD vs. 10 CD-GFD vs. 8 HC	Children	No	Yes	No	FISH Flow citometry	↓ Ratio of *Lactobacillus–Bifidobacterium to Bacteroides–E. coli* ↑ Gram-negative
Collado et al. [31]	8 CD vs. 8 CD vs. 8 HC	Children	No	Yes	Yes	real-time PCR	↑ *Bacteroides, C. leptum, E. coli, Staphylococcus* ↓ *Bifidobacteria*
Di Cagno et al. [32]	7 CD vs. 7 CD-GFD vs. 7 HC	Children	No	No	Yes	real time PCR DGGE	↓ Ratio of cultivable lactic acid bacteria and *Bifidobacterium* to *Bacteroides* and *enterobacteria* ↓ *Lactobacillus*
Ou et al [33]	45 CD vs. 18 HC	Children	No	Yes	No	16S rDNA sequencing	↑ *Haemophilus, Streptococcus, Neisseria*
Schippa et al. [34]	20 CD before and after GFD vs. 10 HC	Children	No	Yes	No	16S rDNA sequencing TTGE	↑ *Bacteroides vulgatus* and *Escherichia coli*
De Palma et al. [35]	24 CD vs. 18 CD-GFD vs. 20 HC	Children	No	No	Yes	FISH flow cytometry	↓ Gram-positive to Gram-negative bacteria ratio ↓ *Bifidobacterium, Clostridium histolyticum, C. lituseburense* and *Faecalibacterium prausnitzii* ↑ *Bacteroides–Prevotella*
Sanchez et al. [36]	20 CD vs. 12 CD-GFD vs. 8 HC	Children	No	Yes	No	DGGE	↑ *Bacteroides dorei* ↓ *Bacteroides distasonis, Bacteroides fragilis/Bacteroides thetaiotaomicron, Bacteroides uniformis,* and *Bacteroides ovatus* ↑ *Bifidobacterium adolescentis Bifidobacterium animalis* subsp *lactis*
Di Cagno et al. [37]	19 CD vs. 15 HC	Children	No	Yes	Yes	DGGE	↓ *Lactobacillus, Enterococcus,* and *Bifidobacteria*
Nistal et al. [38]	10 CD vs. 11 CD-GFD vs. 11 HC	Adults	No	No	Yes	DGGE	↑ *B. bifidum and catenulatum*
Nistal et al. [39]	13 CD vs. 5 CD-GFD vs. 10 HC	Children Adults	No	Yes	No	16SrRNA gene sequencing	↓ *Streptococcus* and *Prevotella*
Sanchez et al. [40]	20 CD vs. 20 CD-GFD vs. 20 HC	Children	No	No	Yes	PCR DNA sequencing	↑ *Staphylococcus epidermidis Staphylococcus haemolyticus* ↓ *S. aureus*
Acar et al. [41]	35 CD vs. 35 HC	Children	Yes	No	No	CRT Bacteria	↓ Salivary mutans streptococci and lactobacilli colonization
De Meij et al. [42]	21 CD vs. 21 HC	Children	No	Yes	No	IS-pro, profiling method	No differences
Sanchez et al. [43]	32 CD vs. 17 CD-GFD vs. 8 HC	Children	No	Yes	No	Colture 16S rRNA gene sequencing	↑ Proteobacteria, Enterobacteriaceae, and Staphylococcaceae ↓ Streptococcaceae, Firmicutes
Wacklin et al. [44]	33 CD (either symptomatic or asymptomatic) vs. 18 HC	Adults	No	Yes	No	16S rRNA gene sequencing	↑ Proteobacteria, such as *Acinetobacter* and *Neisseria*, in patient with GI symptoms. ↓ microbial diversity in GI symptoms or anemia
Cheng et al [45]	10 CD vs. 9 HC	Children	No	Yes	No	qRT-PCR	No differences *Haemophilus* ssp. and *Serratia* ssp. had relatively higher abundance in CD
Francavilla et al. [46]	13 CD-GFD vs. 13 HC	Children	Yes	No	No	16S rRNA gene sequencing	↑ Lachnospiraceae, Gemellaceae, and *Streptococcus sanguinis* Bacteroidetes ↓ *Streptococcus thermophilus*
Wacklin et al. [47]	18 CD-GFD symptomatic vs. 18 CD-GFD asymptomatic	Adults	No	Yes	No	16S rRNA gene sequencing	↑ Proteobacteria ↓ *Bacteroides* and Firmicutes
Giron-Fernandez Crehuet et al. [48]	11 A-CD vs. 11 HC	Children	No	Yes	No	DGGE	*Lactobacillus* genus
D’Argenio et al. [49]	20 A-CD vs. 6 CD-GFD vs. 15 HC	Adults	No	Yes	No	16S rRNA gene sequencing metagenomics	↑ Proteobacteria ↓ Firmicutes and Actinobacteria ↑ *Neisseria* genus (*Neisseria flavescens*)
Quagliariello et al. [50]	40 A-CD vs. 16 HC	Children	No	No	Yes	16S rRNA gene sequencing Quantitative PCR (qPCR)	↓ Firmicutes/Bacteroidetes ratio, ↓ Actinobacteria and Euryarchaeota
Tian et al. [51]	21 CD-GFD vs. 8 RCD vs. 20 HC	Adults	Yes	No	No	16S rRNA gene sequencing	Bacteroidetes (CD > RCD), Actinobacteria (CD < RCD), Fusobacteria (CD > RCD)

A-CD: active celiac disease, CD-GFD: celiac disease on gluten-free diet, GI: gastrointestinal, RCD: refractory celiac disease, HC: healthy controls, FISH: fluorescent in situ hybridization, TTGE: temporal temperature gradient gel electrophoresis, DGGE: denaturing gradient gel electrophoresis; qPCR: quantitative PCR; qRT-PCR: quantitative reverse-transcriptase-PCR; ↓ Decrease; ↑ Increase.

**Table 2:** Main evidence on the use of probiotics in patients with celiac disease.

Author	RCT	Population	Used Strain	Time of Administration	Findings in Probiotics Group
Smecuol et al. [63]	Yes	22 A-CD (12 *probiotic* vs. 10 placebo)	*Bifidobacterium infantis* Natren life start	3 weeks	Improvement in GI symptoms (indigestion, constipation, and gastroesophageal reflux) ↓ Final/baseline IgA tTG and IgA DGP antibody concentration ratios ↑ Serum macrophage inflammatory protein-1β No differences in intestinal permeability No significant changes in cytokines and chemokines production
Pinto-Sánchez et al. [64]	No	24 A-CD no treatment vs. 12 A-CD probiotic treatment vs. 5 CD-GFD	*Bifidobacterium infantis* Natren life start	3 weeks	↓ Paneth cell counts ↓ α-defensin-5
Olivares et al. [65]	Yes	36 A-CD (18 *B. longum* + GFD vs. 18 placebo + GFD)	*Bifidobacterium longum* CECT 7347	3 months	↑ Height percentile ↓ Peripheral CD3^+^ T lymphocytes concentration ↓ TNF-α levels ↓ *Bacteroides fragilis* and Enterobacteriaceae ↑ Harmless to potentially harmful bacteria ratio No differences in GI symptoms
Quagliarello et al. [50]	Yes	40 A-CD children (20 probiotic and 20 placebo) vs. 16 HC	*Bifidobacterium breve strains* (B632 and BR03)	3 months	↑ Actinobacteria Re-establishment Firmicutes/Bacteroidetes ratio.
Harnett et al. [66]	Yes	45 CD-GFD with symptoms (23 probiotic and 22 placebo)	multispecies probiotic VSL#3 (450 billion viable lyophilized bacteria *Streptococcus thermophilus, Bifidobacterium breve, Bifidobacterium longum, Bifidobacterium infantis, Lactobacillus acidophilus, Lactobacillus plantarum, Lactobacillus paracasei,* and *Lactobacillus delbrueckii subsp. Bulgaricus)*	12 weeks	No differences in the fecal microbiota counts No differences in symptoms severity
Klemenak et al. [67]	Yes	49 CD-GFD (24 probiotic and 25 placebo) 18 HC	*Bifidobacterium breve strains* (BR03 and B632)	3 months	↓ TNF-alpha levels (not persistent)
Primec et al. [68]	Yes	40 CD (20 probiotic and 20 placebo) 16 HC	*Bifidobacterium breve strains* (BR03 and B632)	3 months	Negative relationship between Firmicutes and pro-inflammatory TNF-α.
Francavilla et al. [69]	Yes	109 CD-GFD with IBS symptoms (54 probiotic vs. 55 placebo)	mixture of 5 *Lactobacillus casei LMG* 101/37 P-17504 *Lactobacillus plantarum* CECT 4528, *Bifidobacterium animalis subsp. lactis* Bi1 LMG P-17502, *Bifidobacterium breve* Bbr8 LMG P-17501 *Bifidobacterium breve* Bl10 LMG P-17500	6 weeks	Improvement in GI symptoms ↑ *Bifidobacteria* (persistent)

A-CD: active celiac disease; CD-GFD: celiac disease on gluten-free diet; HC: healthy controls; GI: gastrointestinal, IgA: immunoglobulin A; tTG: antitransglutaminase; DGP: deamidated gliadin peptide; TNF: tumor necrosis factor; ↓ Decrease; ↑ Increase.
